# Violet Innovation Grade Index (VIGI): A New Survey-Based Metric for Evaluating Innovation in Analytical Methods

**DOI:** 10.1021/acs.analchem.5c00212

**Published:** 2025-03-26

**Authors:** Adrián Fuente-Ballesteros, Víctor Martínez-Martínez, Ana M. Ares, Silvia Valverde, Victoria Samanidou, José Bernal

**Affiliations:** † Analytical Chemistry Group (TESEA), I. U. CINQUIMA, Faculty of Sciences, 16782 University of Valladolid, 47011 Valladolid, Spain; ‡ Faculty of Sciences and Technology, 421806 Isabel I University, 09003 Burgos, Spain; § Laboratory of Analytical Chemistry, School of Chemistry, 193601 Aristotle University of Thessaloniki, GR 54124 Thessaloniki, Greece

## Abstract

The violet innovation grade index (VIGI) is a pioneering metric designed to evaluate the degree of innovation in analytical methods. This study introduces the VIGI tool (https://bit.ly/VIGItool) and demonstrates its application in assessing the innovative potential of various analytical techniques. VIGI integrates ten distinct criteria—sample preparation and instrumentation, data processing and software, white analytical chemistry and its derivatives, regulatory compliance, materials and reagents, miniaturization, automation, interdisciplinarity, sensitivity, and approach—providing a comprehensive evaluation that complements existing green, blue, and red metrics. Each method is assessed using a survey-based approach, resulting in a star-shaped decagon pictogram that visually represents its innovation score. The VIGI metric was successfully applied to five case studies, revealing insights into the strengths and weaknesses of each method in terms of innovation. Methods incorporating advanced materials, miniaturized devices, and automation scored highly, reflecting their cutting-edge contributions to analytical chemistry. Conversely, methods lacking advanced data processing or interdisciplinary applications scored lower, highlighting areas for potential improvement. This work underscores the importance of prioritizing innovative metrics like VIGI in the development and evaluation of analytical methods to ensure that analytical chemistry remains at the forefront of scientific advancement through more effective and sustainable practices.

## Graphical abstract

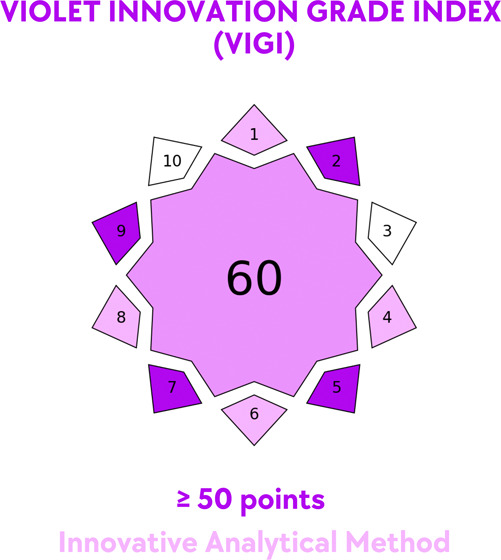

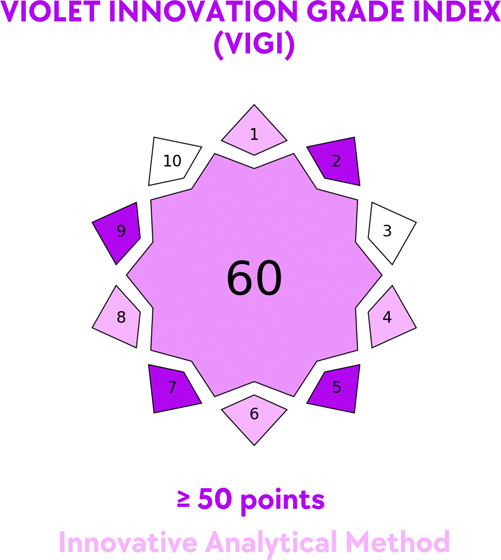

## Introduction

Green chemistry has become a cornerstone in the effort to promote sustainable practices in laboratories. Guided by the 12 principles of green chemistry, this field encourages the development of chemical processes that are more environmentally friendly, economically viable, and socially responsible. These principles serve as a roadmap for chemists across various disciplines, aiming to reduce or eliminate the use of hazardous substances, minimize waste, and lower energy consumption. By embracing these guidelines, chemists have made significant strides in creating processes that are safer for both the environment and human health.^1^ However, although much attention has been given to the greening of synthetic chemistry and industrial processes, the field of analytical chemistry presents its own set of unique challenges and opportunities in the context of sustainability. Since all the principles of green chemistry do not entirely suit the particular challenges of analytical chemistry, 12 principles of green analytical chemistry (GAC) were formulated in 2013,^2^ offering a specific framework for making analytical methods more sustainable.^1^ These principles emphasize the need to reduce the use of hazardous materials, minimize waste, and increase the energy efficiency of analytical procedures. By integrating these principles into practice, GAC aims to foster the development of greener, more sustainable methods for chemical analysis, thereby contributing to the broader goals of green chemistry.^3–5^

In tandem with the development of greener methods, more than 16 metric tools have been developed over the past years to measure the degree of compliance of methods with the GAC concepts.^6^ Some of these metrics include the national environmental methods index (NEMI), analytical eco-scale, analytical method volume intensity (AMVI), green analytical procedure index (GAPI), and analytical greenness calculator (AGREE).^7^ All these metrics take different aspects of the analytical procedure into account to provide the green index of the procedure, and it is highly recommended to research and compare various metrics to determine which is best suited for a particular analytical method. The most commonly used green metrics often do not adequately capture the full scope of method development.^8^ In this regard, white analytical chemistry (WAC) offers a compelling approach with a holistic perspective, incorporating not only environmental, but also analytical and practical aspects.^6^ The 12 WAC principles were introduced as an alternative to the existing 12 GAC principles, emphasizing various criteria that influence ecological aspects (green), the quality and performance of a method from an analytical perspective (red), and its practicality (blue).^9^ However, the WAC principles do not consider the innovative potential of an analytical method, which is a critical aspect in contemporary society, facilitating adaptation to emerging chemical challenges and approaches to complex data analysis.^10^ The measurement of innovation is vital for advancing scientific progress, enhancing efficiency, and reducing costs, as well as promoting environmental sustainability.^11^ Such an evaluation enables laboratories and institutions to maintain competitiveness and adhere to evolving regulations, ensuring that analytical methods are precise, safe, and effective.

This work introduces, for the first time, a visual and rapid survey-based tool designed to evaluate the degree of innovation in analytical methods. The violet innovation grade index (VIGI) is a novel metric that reflects the added value of a method innovation. VIGI serves as a complementary indicator to existing green metrics and other recently developed tools, such as the blue applicability grade index (BAGI).^12^ VIGI works through a straightforward process, assessing the degree of alignment of an analytical method with ten proposed criteria, resulting in a graphical representation of the outcomes. To increase its usability, software was created, with its functionality showcased through its application to different analytical methods. VIGI is primarily intended for chemists engaged in the development and implementation of new analytical methods. The VIGI tool is anticipated to complement existing WAC approaches and contribute to the evaluation of innovation in the field of analytical chemistry. By aligning with the pursuit of more innovative and effective analytical methods, we expect this metric to gain adoption among a diverse audience, thereby advancing the innovation in analytical practices.

## THE CORE ASPECTS OF THE VIGI TOOL

VIGI is a new survey-based metric for evaluating the innovation in analytical methods. It is based on asking 10 questions to the analytical method and answering according to the degree of agreement. All statements begin with the structure “Does the analytical method...?” followed by the application of each of the criteria listed in Table 1. The selection of those main attributes was based on key trends in analytical chemistry. For ease of assessment, three distinct point values are used: 10, 5, and 0. Each score is associated with a specific hue (dark violet #9710EA, light violet #E5B3FE, and white #FFFFFF, respectively), representing from a high level to a low level of innovation. The result of applying VIGI is a violet pictogram of a star-shaped decagon. Its interpretation is similar to that of the analytical greenness calculator (AGREE), analytical greenness metric for sample preparation (AGREEprep) or BAGI,^7,12,13^ and is based on two key parameters. First, the color intensity indicates the degree of innovation: the darker the shade of violet, the more innovative the analytical method. Second, the number in the inner part represents the overall score of the analytical method on a 0–100 scale; the lower this value, the less innovative the method (see Figure 1).

**Table 1:** Detailed Description of the Main Features and Criteria of the VIGI Tool^a^

			Violet	Light violet	White
			10 points	5 points	0 points
Criterion	Parameter	Question: Does the analytical method...?	High innovation	Regular innovation	Low innovation
1	Sample prep. and instrumentation	Apply advanced sample preparation methods (e.g., SPME, DLLME, MEPS, SBSE, FPSE, MAE, LIS, EME, CPME, etc.) and/or techniques (2D chromatography, MS/MS, HRMS, etc.)	Strongly agree	Moderately agree	Disagree
2	Data processing and software	Incorporate new data processing techniques (e.g., AI tools, algorithms, bioinformatics, machine learning, big data, blockchain, statistic advances, simulations, molecular networking, in-silico predictions, etc.)	Strongly agree	Moderately agree	Disagree
3	White analytical chemistry and its derivatives	Consider analytical chemistry principles, metrics or indexes (e.g., AGREE, AGREEprep, MoGAPI, BAGI, RAPI, etc.)	Strongly agree	Moderately agree	Disagree
4	Regulatory compliance	Address problems, needs, and/or recommendations highlighted by relevant organizations, entities, or legislative bodies at the local, national, or international level	Strongly agree	Moderately agree	Disagree
5	Materials and reagents	Utilize innovative materials and/or reagents (e.g., 3D printers, MOFs, graphene, nanostructures, ILs, DES, NADES, SUPRAS, carbon dots, MIPs, etc.)	Strongly agree	Moderately agree	Disagree
6	Miniaturization	Use of miniaturized devices (e.g., portable instruments, smartphones-based applications, sensors, lab-on-a-chip, microcomputers, microfluidic, etc.)	Strongly agree	Moderately agree	Disagree
7	Automatization grade	Integrate automatization (e.g., robotic systems, online analyses, on-flow techniques, etc.)	Strongly agree	Moderately agree	Disagree
8	Interdisciplinarity	Extrapolate to different areas of science and/or industry (e.g., environmental monitoring, pharmaceutical applications, food safety, etc.) and promote collaborations	Strongly agree	Moderately agree	Disagree
9	Sensitivity	Improve LOQ and LOD values compared to previous methods (e.g., achieving nano/pico levels)	Strongly agree	Moderately agree	Disagree
10	Approach	Introduce a new approach for research (e.g., previously unexplored application, new analytes for study, new theoretical frameworks, hot topics, etc.)	Strongly agree	Moderately agree	Disagree

a Legend: AI, Artificial Intelligence; AGREE, Analytical Greenness Calculator; AGREEprep, Analytical Greenness Metric for Sample Preparation; BAGI, Blue Applicability grade Index; CPME, Capsule Phase Microextraction; DES, Deep Eutectic Solvents; DLLME, Dispersive Liquid–Liquid Microextraction; EME, Electromembrane Extraction; FPSE, Fabric Phase Sorptive Extraction; HRMS, High-Resolution Mass Spectrometry; IL, Ionic Liquid; LIS, Lab-in-Syringe; LOD, Limit of Detection; LOQ, Limit of Quantification; MAE, Microwave-Assisted Extraction; MEPS, Microextraction by Packed Sorbent; MIP, Molecularly Imprinted Polymers; MOF, Metal–Organic Frameworks; MoGAPI, Modified Green Analytical Procedure Index; MS/MS, Tandem Mass Spectrometry; NADES, Natural Deep Eutectic Solvents; RAPI, Red Analytical Performance Index; SBSE, Stir Bar Sorptive Extraction; SPME, Solid-Phase Microextraction; SUPRAS, Supramolecular solvents.

**Figure 1:**
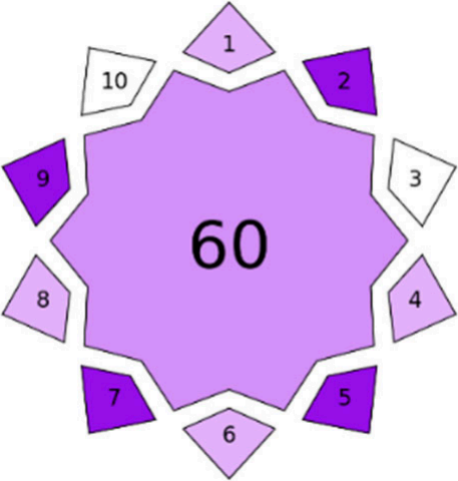
VIGI index pictogram example. The violet intensity indicates the degree of innovation, and the number in the inner part represents the overall score of the analytical method on a 0–100 scale.

Additionally, a threshold value of 50 or higher was defined for a method to be considered innovative. It is important to note that this value is somewhat subjective as the user must coherently justify the resulting score. The ten segments of the pictogram are linked to different performance criteria, each with equal weight in the final score, and the color tone displayed in the central field represents the overall contribution of all the parameters involved. The use of this questionnaire is straightforward, and it is available as a free, open-source desktop resource. This resource has been designed inspired in the AGREEmip tool and implemented starting from the work of Wojnowski et al.,^14^ and it can be downloaded using the link https://bit.ly/VIGItool and following the instructions displayed in Table 2. The VIGI metric tool evaluates the innovation grade of an analytical method by considering the ten primary attributes outlined in the subsequent sections.

**Table 2:** Instructions for the Proper Use of VIGI Tool

Step	Instructions
1	Click or copy the following link (https://bit.ly/VIGItool) in your browser to download a compressed folder with the software
2	Unzip the folder, where you will find two files (the software and a folder with the logo). Both need to be kept together in the same folder for VIGI to work properly. If you need a software to unzip the folder, you can download and install 7-Zip from https://www.7-zip.org/
3	Double-click on the “VIGI software” or run it as administrator (right-click using the mouse). In case the downloaded file triggers an antimalware warning message, it can be safely ignored
4	Select the most appropriate options for each of the 10 criteria. VIGI is a metric for evaluating the innovation of analytical methods in chemistry. Each method is assessed using a survey-based approach, resulting in a star-shaped decagon pictogram that visually represents its innovation score. Methods are rated on a violet scale based on 10 attributes, where higher scores and darker colors indicate a greater degree of innovation. A threshold value of 50 or higher has been defined for a method to be considered innovative
5	Once finished, click on File > Save Image. The figure in PNG format is ready to be included in your manuscript
6	Do not forget to cite us in your reference list: click on About > Info. If you encounter any issue or problems with the software, please contact us

### Sample Preparation and Instrumentation

The first attribute in the VIGI metric evaluates the innovation of analytical methods based on the advanced techniques employed in both sample preparation and the instrumentation used for analysis. This feature is crucial because innovation in these areas can significantly enhance the efficiency, precision, and sustainability of an analytical method. Sample preparation is a fundamental step in any analytical method, as it directly affects the quality of the results obtained.^15^ The VIGI metric positively values (violet color; 10 points) methods that employ advanced sample preparation techniques such as solid-phase microextraction (SPME),^16,17^ dispersive liquid–liquid microextraction (DLLME),^18,19^ microwave-assisted extraction,^20^ lab-in-syringe (LIS),^21,22^ electromembrane extraction,^23^ or capsule phase microextraction (CPME).^24^ Innovative sample preparation methods not only reduce the time invested, but also minimize solvent consumption and exposure to toxic solvents.^25,26^ Moreover, these approaches can allow for the preconcentration of analytes at trace levels, which is especially important in fields such as food and environmental analysis.^27^

Instrumentation is also a key factor in evaluating the innovation of a method. The VIGI metric assigns higher scores to methods that utilize state-of-the-art instrumentation. These technologies, though energy-intensive and costly, enable more-detailed and precise analysis while also being more efficient in terms of time and resources. In contrast, the use of simpler instrumentation available in most laboratories, while it is categorized as practical,^12^ is penalized by VIGI due to its lower capacity to innovate and introduce new analytical approaches. This aspect is rooted in the VIGI metric’s emphasis on not just the basic functionality of instrumentation but also its ability to address complex analytical challenges and adapt to new scientific and regulatory demands. Advanced techniques like tandem mass spectrometry not only enhance the selectivity and sensitivity of the analysis but also enable the simultaneous identification of multiple compounds in complex matrices, something not as easily achievable with more conventional instrumentation. Furthermore, the VIGI metric recognizes that investing in advanced technologies can lead to greater long-term efficiency. Although the initial costs and energy consumption are high, the ability to perform faster and more precise analyses can result in lower reagent use, fewer experimental repetitions, and a reduction in the total sample processing time, which are crucial in high-throughput environments such as the pharmaceutical industry or environmental research.

### Data Processing and Software

Data processing is an essential aspect of any modern analytical method, and its importance has grown significantly with technological advancements. In the context of the VIGI metric, this attribute evaluates the ability of an analytical method to incorporate innovative data processing techniques that optimize the quality and accuracy of the results. The adoption of tools such as artificial intelligence (AI),^28^ machine learning,^29^ and advanced algorithms^30^ allows for more efficient and precise analysis of large data volumes, identifying patterns and correlations that would be difficult to detect using traditional methods. As the volume of data generated in laboratories continues to grow, the management of big data has become fundamental. The ability to create, store, and process databases is essential for the development and advancement of analytical methods.^31^ Furthermore, blockchain technology can provide unparalleled traceability and security in the management of analytical data, ensuring the integrity of the results and complying with the strictest regulations.^32^ In the field of drug discovery, in-silico approaches enable the rapid screening and modeling of potential drug candidates, significantly reducing the time and cost associated with traditional experimental methods. By simulating molecular interactions, predicting pharmacokinetics, and identifying potential off-target effects early in the process, in-silico approaches streamline the development pipeline, allowing researchers to focus on the most promising compounds. This not only accelerates the discovery of new drugs but also enhances their safety and efficacy by refining candidate selection before moving to costly and time-consuming laboratory and clinical testing stages.

The software used in data processing is another key component in the VIGI metric. This attribute focuses on the method’s ability to integrate advanced software that facilitates data management, analysis, and visualization. Software that offers an intuitive interface and advanced automation options allows users to perform complex analyses with less effort and reduces the possibility of human errors. Additionally, the ability of software to integrate with various platforms and instruments is essential for ensuring an efficient workflow in the laboratory. Software that incorporates simulation and prediction capabilities, such as molecular networking, adds significant value to the analytical process by enabling the anticipation of results and optimization of experimental conditions before costly and time-consuming trials. The use of advanced data processing techniques and optimized software can significantly reduce operational costs and improve data-driven decision-making. The ability to process and analyze large volumes of data in real time is particularly valuable in sectors, such as pharmaceuticals and petrochemicals. Analytical methods that incorporate advanced data processing technologies and software are not only more innovative but also more adaptable to different types of analyses and matrices, allowing a method to be versatile and applicable in multiple contexts, which maximizes its utility and return on investment.

### White Analytical Chemistry and Its Derivatives

The WAC attribute in VIGI evaluates the innovation of analytical methods by considering the foundation. In this regard, the metric takes into account whether the work considers some of the recently developed principles, metrics, and indexes such as AGREE,^7^ AGREEprep,^33^ complementary green analytical procedure index (ComplexGAPI),^34^ modified complementary green analytical procedure index (MoGAPI),^35^ BAGI,^12^ or red analytical performance index (RAPI),^36^ among others. This approach combines environmental sustainability with practical applicability and performance, providing a comprehensive assessment of the analytical method.

Green chemistry promotes the development of techniques that minimize the use of hazardous substances, reduce waste generation, and optimize energy consumption. These principles are increasingly important in analytical research due to stricter environmental regulations and the growing need for sustainable practices. The recently developed BAGI approach allows for the identification of a method’s strengths and weaknesses in terms of practicality and applicability.^12^ For example, by using the VIGI metric, an analytical method that incorporates GAC principles and/or the BAGI tool receives a higher score and a more intense color as it significantly contributes to sustainability and practicality. Encouraging the development of methods that are both sustainable and efficient leads to the recognition of those that integrate green and blue principles as more innovative, and they are valued more highly in terms of their impact and applicability in analytical chemistry. Notably, more and more researchers are considering both aspects in their method development.^37,38^ By fostering a holistic approach that addresses both environmental and practical concerns, the VIGI metric ensures that new methods are not only technically advanced but also aligned with contemporary needs for responsible and accessible scientific practices. This linkage among sustainability, applicability, and innovation underscores the critical role of the WAC attribute in guiding the future of analytical method development.

### Regulatory Compliance

Regulatory compliance is key to assessing the innovation of analytical methods by assessing how well these methods align with the problems, needs, and recommendations highlighted by relevant organizations, entities, or legislative bodies at the local, national, and international levels. This aspect of the VIGI metric ensures that the method not only achieves technical excellence but also adheres to the standards and guidelines set by authoritative bodies, making it both effective and legally compliant. Meeting regulatory standards is necessary for a method to be accepted and applied across various sectors, such as environmental monitoring, pharmaceuticals, and food safety. Regulatory bodies such as the Environmental Protection Agency, the European Medicines Agency, and the Food and Drug Administration set stringent requirements for accuracy, precision, and reliability of analytical methods. These requirements are designed to ensure that the methods produce results that are not only scientifically valid but also legally defensible. Thus, the VIGI metric takes into account whether a method addresses certain standards, guidelines, or directives, reflecting its ability to innovate within an existing regulatory framework.

Incorporating regulatory compliance into the VIGI metric also highlights the method’s responsiveness to emerging global challenges. As environmental regulations become more stringent due to concerns about pollution and climate change, analytical methods must evolve to meet these new requirements. A method that can detect trace pollutants with high sensitivity and specificity, while also being compliant with current environmental legislation, would score higher on the VIGI metric. Moreover, the VIGI metric may evaluate whether the method can be adaptable to changes in regulatory frameworks. Regulatory standards are not static; they evolve as new scientific knowledge becomes available. A method that is flexible and can be easily adapted to comply with the updated regulations is considered more innovative. For instance, analytical methods that can be modified to meet new thresholds for contaminant levels in water or food, as mandated by international agreements, are given higher scores in the VIGI metric. This adaptability is crucial for maintaining the method’s relevance and utility in the face of changing legal and regulatory landscapes.

To the best of our knowledge, VIGI stands out as the first metric to explicitly incorporate regulatory compliance as a key factor in evaluating the innovation of analytical methods. This inclusion is significant because it bridges the gap between scientific innovation and regulatory acceptance. By ensuring that methods are both innovative and compliant with regulatory standards, the VIGI metric not only promotes the development of cutting-edge analytical techniques but also facilitates their adoption and implementation in real-world settings where legal and regulatory considerations are paramount.

### Materials and Reagents

The fifth attribute evaluates the innovation of analytical methods based on the incorporation of advanced and novel substances. In the rapidly evolving field of analytical chemistry, the selection of these components can significantly influence a method’s effectiveness and innovativeness. While traditional options remain reliable, they may not offer the same level of performance or environmental benefits as newer alternatives. The VIGI metric encourages the adoption of cutting-edge materials and reagents, such as metal–organic frameworks (MOFs),^39^ graphene,^40^ nanostructures,^41^ ionic liquids (ILs),^42^ and deep eutectic solvents (DES),^43^ which are at the forefront of modern chemistry. These innovative choices not only enhance the sensitivity and specificity of analytical methods but also promote more sustainable practices. For instance, the use of MOFs in sample preparation and separation processes offers unparalleled surface area and tunable pore structures, allowing for more efficient capture and analysis of target compounds.^44^ Similarly, graphene and other nanostructures enhance the conductivity and sensitivity of sensors, enabling the detection of trace analytes with higher precision.^45^ Lastly, ILs and DES, known for their low volatility and tunable properties, provide greener alternatives to traditional solvents, aligning with the principles of green chemistry.^46^

The VIGI metric assigns higher scores to methods that incorporate these advanced materials and reagents, recognizing their role in pushing the boundaries of analytical science. A method that utilizes common, commercially available reagents might be considered practical, but it may not score as highly in terms of innovation compared with a method that employs novel, synthesized materials specifically designed to address complex analytical challenges. Another example is the use of 3D-printed devices in sample handling^47^ or the integration of molecularly imprinted polymers (MIPs) for selective analyte recognition can significantly elevate a method’s innovative profile.^14,48^ Additionally, the adaptability and scalability of these materials are considered to be within the VIGI framework. Methods that can easily integrate advanced materials into routine laboratory procedures without requiring extensive reconfiguration of existing equipment or protocols are rated more favorably. By emphasizing the use of innovative materials, the VIGI metric not only promotes the development of high-performance analytical techniques but also fosters the adoption of greener, more-efficient solutions in the field of analytical chemistry.

### Miniaturization

The sixth attribute is related to miniaturization, specifically the use of miniaturized devices (e.g., portable instruments, smartphone-based applications, sensors, lab-on-a-chip, microcomputers, microfluidics, etc.). Miniaturization is intrinsically linked to innovation because it fundamentally alters the way analytical processes are designed, conducted, and applied in real-world scenarios. The integration of miniaturized devices into analytical methods not only represents technological advancement but also reflects a method’s adaptability, efficiency, and ecofriendliness in response to contemporary challenges in various fields.

Traditional analytical methods often rely on large, stationary instruments that require significant infrastructure and specialized operators. In contrast, miniaturized devices, such as portable instruments and smartphone-based applications, enable these processes to be conducted in the field, in real time, and with minimal infrastructure.^49^ This shift toward portability is a clear marker of innovation because it expands the potential use cases of analytical methods, making them applicable in remote, resource-limited settings where conventional laboratory setups are impractical. The ability to conduct on-site testing using portable sensors enables immediate data collection and analysis, which proves particularly valuable for the effective monitoring of environmental pollutants.^50^ Furthermore, the use of smartphone-based applications in analytical methods exemplifies how miniaturization can drive innovation by leveraging existing technologies in new ways. These applications often integrate sophisticated sensors and data processing algorithms into devices that are already widely available and familiar to users. This not only reduces the cost and complexity associated with specialized analytical equipment, but also opens up new possibilities for crowd sourced data collection and real-time monitoring by nonexperts.^51^ The innovation here lies in the method’s ability to transform everyday technology into a powerful analytical tool, democratizing access to high-quality data and empowering a broader range of users to engage in scientific inquiry.

Lab-on-a-chip technologies and microfluidic devices are perhaps the most illustrative examples of how miniaturization is synonymous with innovation. These systems condense entire laboratory processes into a single, compact chip, allowing for the automation of complex analytical procedures that would traditionally require multiple steps and significant manual intervention. The innovation is evident in the way these technologies enhance the efficiency, speed, and accuracy of analyses, often reducing what would be hours or days of work in a traditional laboratory to mere minutes.^52^ Moreover, the precision afforded by microfluidic devices—capable of manipulating minuscule fluid volumes with exceptional accuracy—enables the development of new types of assays and tests that were previously impossible, further pushing the boundaries of what can be achieved in analytical chemistry.^53^ In addition to improving efficiency, miniaturization fosters innovation by enabling the development of sustainable and eco-friendly analytical methods. By reducing the number of reagents and samples required, miniaturized devices minimize waste generation and lower the environmental impact of analytical processes. This aligns with the growing emphasis on GAC and sustainable practices within the scientific community. The ability to achieve high-performance results with a minimal environmental footprint is a significant innovation, as it addresses the dual challenge of maintaining analytical rigor while adhering to principles of sustainability.

Miniaturized devices also drive innovation by facilitating the creation of highly specialized and tailored analytical methods. The small size and modularity of these devices allow them to be easily customized and adapted to specific analytical needs, whether that involves the detection of particular analytes in challenging matrices or the development of point-of-care diagnostic tools.^54^ This adaptability is a hallmark of innovative analytical methods, as it demonstrates a method’s capacity to evolve and meet the ever-changing demands of various scientific fields. The degree to which a method incorporates miniaturized devices is, therefore, a direct reflection of its innovative potential as it embodies the cutting edge of technological development and its practical application in solving contemporary scientific challenges.

### Automatization Grade

The seventh attribute of the VIGI metric measures the degree of innovation of an analytical method by evaluating the automation integrated into the process. This attribute is directly related to the method’s ability to reduce human intervention, which enhances precision and reproducibility and improves the overall efficiency of the analytical process. Automation, when integrated into analytical methods, represents a significant technological advancement, as it allows processes to become more consistent, faster, and less prone to human error. The implementation of automated systems enables analytical procedures to be carried out continuously and without interruptions, which not only improves the speed of analysis but also allows for the handling of a larger volume of samples in a shorter period. This is particularly valuable in laboratories where time is a critical factor and where the demand for analysis is high. A method that can process more samples in less time without compromising the quality of the results is clearly more innovative and efficient, making it more attractive. Moreover, automation reduces the variability associated with manual intervention, leading to greater reproducibility of the results. It is also associated with enhanced laboratory safety. By reducing the need for direct handling of hazardous chemicals, automated methods better protect operators, thereby lowering the risk of exposure and accidents.

Sample preparation is a fundamental step in achieving reliable and accurate analytical outcomes as it ensures selective enrichment, purification, and the elimination of interfering substances from the matrix. However, these procedures are often time-consuming and prone to errors. Automating the sample preparation process significantly enhances throughput, precision, and accuracy while also minimizing the health risks associated with handling hazardous chemicals or biological materials.

Two prevalent methods for automating sample preparation, such as chromatography and mass spectrometry, are robotic systems and on-flow techniques. Robotic systems offer a flexible automation solution capable of executing various chemical tasks with their movable components. A wide range of commercial options are available, and open-source prototyping has made it more accessible and cost-effective to develop and implement lab-based robotic systems. On-flow techniques include various methods that rely on pumps and valves, with column-switching being particularly effective for directly injecting raw samples and seamlessly integrating extraction, preconcentration, and separation steps online.^55^ Rodríguez-Maese et al.^56^ conducted a literature review on several flow analysis techniques to determine and monitor manganese in environmental water samples. They emphasized that automation through flow analysis techniques offers advantages such as significantly increasing sample processing capacity and reducing time and reagent usage, resulting in cost savings and minimized waste production, thereby aligning with the principles of GAC.

### Interdisciplinarity

Interdisciplinarity refers to the ability of a method to be effectively applied across multiple scientific and industrial disciplines, thereby promoting collaborations. This attribute is a key indicator of the method’s versatility and scope, making it a powerful tool in various areas such as environmental monitoring, pharmaceutical industry, and food safety, among others. The ability of an analytical method to be used in different fields is a clear sign of its adaptability and robustness. A method that can be applied both in the detection of contaminants in water samples and in the analysis of active compounds in pharmaceutical products demonstrates great flexibility. This flexibility not only reflects the method’s technical solidity but also suggests that it can meet the rigorous requirements of various industries.

Analytical method interdisciplinarity is frequently associated with its capacity to solve complex problems that require a multifaceted approach. For instance, the integration of new materials and innovative sensing platforms in microfluidic systems to address real-world challenges in diagnosing various diseases in point-of-care settings.^57^ Extrapolating to different areas also implies that the method has been designed or can be adapted to handle various matrices. This capability not only broadens its applicability but also enhances its relevance in situations where analytes need to be detected in complex and varied matrices. A method that can be applied across multiple disciplines is typically supported by advanced technologies and processes that allow it to be adapted to different requirements and standards. What’s more, interdisciplinarity is pivotal in promoting collaborations among researchers from different fields. A method that can be applied across various disciplines facilitates the convergence of experts from different areas, fostering an exchange of knowledge and experiences that can lead to new ideas and innovative approaches. Interdisciplinary collaborations enrich the method development and open new lines of research and applications in other fields, thereby expanding the method’s impact and scope.^58^ The issue of emerging contaminants such as microplastics is particularly noteworthy. Microplastics do not easily fit within traditional risk-based regulatory frameworks due to their persistence and extreme diversity, resulting in high levels of uncertainty in hazard and exposure estimates. Due to these serious complexities, addressing the impacts of microplastics requires open collaboration between scientists, regulators, and policymakers.^59^

Lastly, the potential for a method to be adopted across various scientific and industrial areas also implies greater acceptance and use by the global scientific community. This enhances the method’s visibility while simultaneously fostering interdisciplinary collaboration, which can pave the way for new innovations and applications. Therefore, interdisciplinarity is a key indicator of an innovative analytical method, highlighting its potential to make significant contributions across multiple fields of science and industry and promoting collaboration among researchers for the advancement of scientific knowledge.

### Sensitivity

The ninth attribute measures the degree of innovation of an analytical method in terms of its ability to improve the limits of quantification (LOQ) and detection (LOD), compared to previous methods. This attribute is crucial for determining how advanced a method is in detecting analytes at extremely low concentrations. Analytical method sensitivity refers to its ability to detect and quantify the smallest possible amount of a substance in a given sample. Improving the LOQ and LOD values is an indicator that the method has been optimized to recognize analytes at much lower levels than could previously be detected. This capability is particularly important in fields such as pharmacology, where the precise detection of trace compounds can be critical to the efficacy of a medication, or in environmental monitoring, where the identification of contaminants at nano or pico levels can be crucial for assessing public health risks.^60^

Advances in the sensitivity of analytical methods are achieved through various strategies, such as improved instrumentation, the optimization of experimental conditions, and the development of new sample preparation techniques that minimize background noise and increase the signal-to-noise ratio.^61^ A method capable of detecting nanograms or picograms per liter or gram demonstrates a degree of innovation that enables its use in applications requiring high precision and accuracy in the detection of analytes in complex matrices.^62^

The potential of a method to reach trace levels is a reflection of technological advances in analytical instrumentation. The development of high-resolution mass spectrometry, advanced chromatography, and sophisticated preconcentration techniques has been key to achieving these improvements in sensitivity.^63^ The implementation of these advances in an analytical method not only makes it more sensitive but also more competitive in an environment where precision and accuracy are increasingly valued.

### Approach

The final attribute assesses the degree of innovation in an analytical method by evaluating its capacity to introduce novel approaches in scientific research. This attribute is decisive in determining to what extent a method not only contributes new data or results but also drives the development of new perspectives and applications within the field of analytical chemistry. A method that introduces a new approach can do so in various ways, such as by exploring applications that have not been studied. This includes, for instance, the detection of new analytes that were not previously considered relevant or the study of matrices that have not yet been analyzed. The introduction of these novel elements not only broadens the method’s scope of application but can also open new lines of research, generating a significant impact on the discipline. Moreover, an innovative approach may involve the development of new theoretical frameworks that provide a deeper or different understanding of the phenomena being analyzed. These theoretical frameworks allow for a richer interpretation of the data obtained and can also influence the design of future experiments and the approach to complex problems in analytical chemistry. The importance of introducing new approaches in analytical chemistry is particularly relevant when dealing with cutting-edge topics such as those defined by the European Chemicals Agency (ECHA). These “hot topics” include, among others, emerging contaminants, microplastics, and endocrine disruptors, which have recently been the focus of some studies.^37,64^ Addressing these fields means not only staying abreast of the latest developments but also actively contributing to the evolution of scientific knowledge, positioning research at the forefront of analytical chemistry, and offering solutions that can have a real impact on society and the environment.

## PRACTICAL EXAMPLES: HOW CAN VIGI BE APPLIED TO ANALYTICAL METHODS?

As a representative example, five practical cases were selected to which this new tool has been applied. This comparative analysis aims to identify the strengths and weaknesses of each method, providing a comprehensive view of the degree of innovation, according to our criteria. The resulting VIGI pictograms for these methods are shown in Table 3.

**Table 3:**
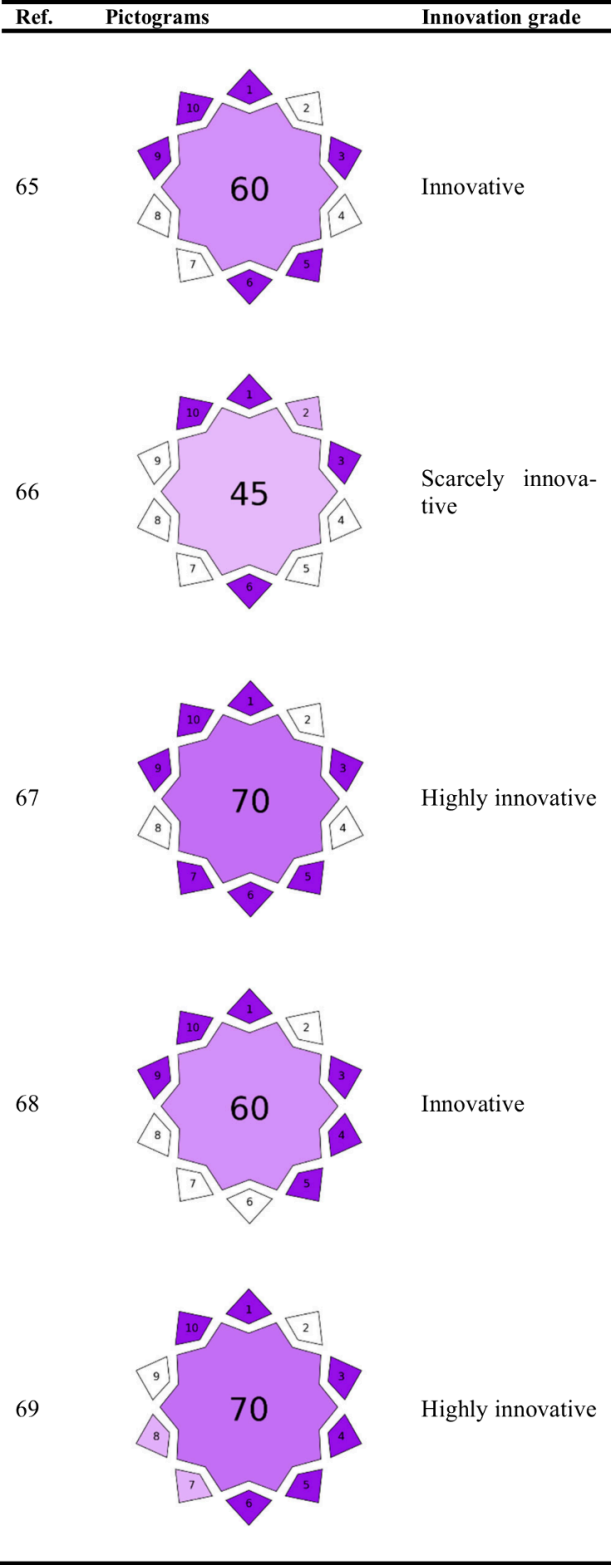
VIGI Pictograms for the Five Selected Analytical Methods

The first article by Manousi et al.^65^ addressed the development of an analytical methodology that combined CPME with liquid chromatography–mass spectrometry (LC-MS) for the detection of phosphodiesterase-5 inhibitors in biological samples. Regarding the first parameter, related to advanced sample preparation and instrumentation, the article showed outstanding results. The implementation of CPME represented a modern and efficient microextraction technique that significantly improved the sample preparation process by integrating the filtration and stirring mechanisms directly into the device. Therefore, a score of 10 points was assigned, indicating a high level of innovation in this aspect. Concerning the use of advanced data processing techniques and software, the study did not show evidence of incorporating sophisticated tools. Regarding the GAC principles, the developed method demonstrated a significant commitment to sustainability principles. The method made minimal use of organic solvents and reduced waste generation, in addition to employing the ComplexGAPI index to evaluate and ensure the ecological nature of the procedure, along with the BAGI tool, which demonstrated the practicality of the method. Additionally, microextraction capsules could be reused at least 25 times for both urine and serum samples, and “green solvents” were employed. Later, the review did not explicitly address how the developed method aligned with regulatory guidelines from local, national, or international entities. For the fifth parameter, the article stood out by utilizing sorbents functionalized with ILs and Carbowax 20M. These advanced materials enhanced the selectivity and efficiency of the extraction process, representing a novel approach to the design of stationary phases. Therefore, a score of 10 points was assigned. Considering the miniaturization aspect, the method successfully employed CPME, involving small-sized and highly efficient extraction devices. This miniaturization reduced sample and solvent consumption and facilitated the application of the method in resource-limited settings. For the eighth parameter, the study primarily focused on the bioanalytical and pharmaceutical fields without exploring or discussing potential applications in other fields, such as environmental, food safety, or industrial sectors. By contrast, the study demonstrated significant improvements in the LOD/LOQ values, achieving levels on the order of ng/mL, compared to previous methods. The ability to detect very low concentrations of analytes in complex samples underscored the method’s effectiveness and precision. Finally, in relation to the introduction of a novel approach to research, the study presented a unique combination of CPME with IL-functionalized sorbents for the detection of specific inhibitors, an approach that had not been widely explored previously. This innovative method offered new perspectives and possibilities for sample preparation and bioanalytical analysis. Adding up the scores assigned to each parameter, the analytical method described in the paper achieved a total of 60 points out of 100. This result indicated that the method showed a considerable level of innovation, particularly in key aspects, such as sample preparation, green chemistry, and the use of innovative materials, miniaturization, sensitivity, and novel approaches.

The second article^66^ developed a novel method for investigating wine aging, showcasing a high degree of innovation by utilizing the advanced SPME-Arrow technique. This approach represents a cutting-edge method in sample analysis, offering significant advancements in the precision and resolution. However, the study only partially incorporated advanced data processing techniques. Although chemometric tools were employed, the absence of more-sophisticated data analysis methods resulted in a score of 5 points. In terms of the environmental impact, the method was evaluated using the GAPI and BAGI indices. The design of the SPME-Arrow method inherently supports miniaturization, enabling efficient extraction with minimal sample sizes. However, the method fell short in the automation parameter as the study did not implement any automated systems for sample handling or analysis, relying instead on manual operations. Additionally, the interdisciplinarity of the method was limited, specifically applied to wine aging. To the best of the authors’ knowledge, there are no other reports on the use of SPME-Arrow for the extraction of volatile compounds in wine to investigate aging. The overall score for the method based on the VIGI metric is 45/100. This score reflects a method that is innovative in specific technical aspects but could benefit from broader applicability and integration into other fields to increase its overall impact and utility in the scientific community.

The third article^67^ explored several facets of analytical method innovation. In the area of sample preparation and instrumentation, it scored highly by introducing a novel liquid-phase microextraction technique using natural deep eutectic solvents (NADES) within a LIS platform. This modern approach enhanced the sensitivity and efficiency in toxic metal detection. However, the method did not incorporate advanced data processing techniques. The applicability of the proposed method was assessed by using the BAGI metric, and its greenness was evaluated through the GAPI and AGREE tools. The method’s use of innovative materials and reagents was highlighted by the employment of NADES, which provided an eco-friendly alternative to traditional organic solvents. The LIS platform’s miniaturized design allowed for efficient sample processing and reduced environmental impact, contributing to the method’s strong performance in this area. Additionally, the incorporation of a fully automated flow-batch system significantly enhanced the efficiency and precision of the analysis, reducing human intervention and the potential for error. The method also displayed high sensitivity with lower LOD/LOQs. Moreover, the combination of LIS with NADES for the first time in metal determination via flame atomic absorption spectrometry represents a significant advancement in analytical methodology. Overall, the method described in this article achieved a total score of 70 out of 100 points. This high score indicated that the method was highly innovative, particularly in sample preparation, green chemistry, miniaturization, automation, sensitivity, and the introduction of new approaches.

The fourth case study^68^ focuses on a green analytical method for determining 14 bisphenols in bee pollen. The authors proposed and evaluated microextraction using supramolecular solvents, which are considered a promising alternative to conventional solvents/sample treatments, especially for their ability to significantly reduce phospholipid-based matrix effects. In this context, an assessment of the sustainability of the proposed sample treatments was conducted using AGREE, AGREEprep, and ComplexGAPI. Moreover, the research adheres to European food safety standards, particularly concerning bisphenol S, demonstrating its alignment with international regulatory frameworks, which contributes positively to its evaluation. Additionally, potential theoretical hazards associated with bee pollen samples containing quantifiable levels of bisphenols were evaluated through a risk assessment. The LOD and LOQ values were much lower than the limits established by legislation and lower than the LODs and LOQs obtained in previous studies, demonstrating the excellent sensitivity of the proposed method. Lastly, only one other study dedicated to the analysis of bisphenols in pollen has been published, highlighting the innovative nature of this method and its application to an unexplored matrix. Thus, a VIGI score of 60 was assigned to the method, demonstrating its innovation.

The latest article^69^ described the extraction of contaminants from environmental waters and urine using DLLME. The method scored highly in criteria 1, 5, and 6 due to the combination of a microextraction technique with DES, which reduced hazardous waste and energy consumption, as emphasized by the AGREEprep metric. The focus on emerging contaminants, such as bisphenol A, which are regulated under strict standards, positively contributed to its evaluation. While some automation was present through the use of autosamplers in the HPLC system, much of the process required manual handling. The method demonstrated versatility in being applied to contaminants in complex environmental (sea and wastewater) and biological (urine) samples. Moreover, the study characterized for the first time different eutectic mixtures, introducing a novel approach in analytical chemistry. The BAGI score assigned to the method was 70, reflecting its degree of innovation.

To sum up, VIGI is a novel index designed to efficiently assess the innovation of an analytical method. It serves as a complementary tool to existing green and blue assessment frameworks within the context of WAC. Unlike conventional green metrics that primarily focus on environmental sustainability, VIGI integrates an additional layer by evaluating the innovative potential of analytical methods, bridging the gap between green chemistry principles and practical applicability. A key feature of VIGI is the score displayed at the center of its pictogram, allowing for direct comparison of an analytical method’s innovation level with other methods, thereby facilitating the identification of strengths and areas for improvement. The greatest advantage of VIGI is its simplicity and ease of application, which has been achieved through the development of a user-friendly desktop application (https://bit.ly/VIGItool) that enables rapid evaluation of an analytical method’s innovation grade.

## Conclusions

The development and application of VIGI provided a comprehensive and valuable tool for assessing the innovation level of analytical methods. The VIGI metric, which integrated ten distinct criteria, offered a holistic evaluation that complemented existing green, blue, and red metrics. This study demonstrated the utility of VIGI through various case studies, highlighting its ability to pinpoint the innovative strengths and weaknesses of analytical methods. The introduction of VIGI as a survey-based tool added a new dimension to the evaluation of analytical methods, emphasizing the importance of innovation alongside sustainability and practicality. By adopting VIGI, laboratories and institutions will be better equipped to navigate the evolving landscape of analytical chemistry, ensuring their methods were not only environmentally sound and practically feasible but also at the forefront of scientific advancement. This approach aligned with the broader goals of analytical chemistry principles, metrics, and indexes, fostering the development of more effective, sustainable, and innovative analytical practices. The key advantage of VIGI lies in its ability to systematically assess and visualize the innovative potential of analytical methods, making it an indispensable tool for advancing scientific progress in the field.

## ABBREVIATIONS

AGREE: analytical greenness calculator
AGREEprep: analytical greenness metric for sample preparation
AMVI: analytical method volume intensity
BAGI: blue applicability grade index
CPME: capsule phase microextraction
DES: deep eutectic solvents
DLLME: dispersive liquid–liquid microextraction
GAC: green analytical chemistry
ComplexGAPI: complementary green analytical procedure index
ILs: ionic liquids
LC-MS: liquid-chromatography–mass spectrometry
LIS: lab-in-syringe
LOD: limits of detection
LOQ: limits of quantification
MIPs: molecularly imprinted polymers
MOFs: metal–organic frameworks
MoGAPI: modified green analytical procedure index
NADES: natural deep eutectic solvents
NEMI: National Environmental Methods Index
RAPI: Red Analytical Performance Index
SPME: solid-phase microextraction
VIGI: Violet Innovation Grade Index
WAC: white analytical chemistry
